# Supplementary material for: Short dual antiplatelet therapy duration after percutaneous coronary intervention in high bleeding risk patients: Systematic review and meta-analysis
Source: PLoS One. 2023 Sep 1;18(9):e0291061. doi: 10.1371/journal.pone.0291061 (PMC10473507; doi:10.1371/journal.pone.0291061)
Supplement: S1 Appendix — (DOCX) [file pone.0291061.s002.docx]

**S1 Appendix. Database search strategy**

| **MEDLINE (inception to August 18, 2022)** | **Embase (2019 to August 18, 2022)** | **CENTRAL (2019 to August 18, 2022)** |
| --- | --- | --- |
| 1. percutaneous coronary intervention.mp. or Percutaneous Coronary Intervention/ 2. high bleed* risk.mp. 3. Platelet Aggregation Inhibitors/ or antiplatelet.mp. 4. Platelet Aggregation Inhibitors/ or p2y12.mp. 5. clopidogrel.mp. or Clopidogrel/ 6. prasugrel.mp. or Prasugrel Hydrochloride/ 7. ticagrelor.mp. or Ticagrelor/ 8. 3 or 4 or 5 or 6 or 7 9. 1 and 2 and 8 10. limit 9 to "therapy (maximizes specificity)" | 1. percutaneous coronary intervention.mp. or percutaneous coronary intervention/ 2. high bleed* risk.mp. 3. dual antiplatelet therapy/ or antiplatelet.mp. 4. p2y12.mp. 5. acetylsalicylic acid plus clopidogrel/ or clopidogrel/ or clopidogrel.mp. 6. prasugrel.mp. or prasugrel/ 7. ticagrelor.mp. or ticagrelor/ 8. 3 or 4 or 5 or 6 or 7 9. 1 and 2 and 8 10. limit 9 to "therapy (maximizes specificity)" | 1. percutaneous coronary intervention.mp. or Percutaneous Coronary Intervention/ 2. high bleed*.mp. 3. antiplatelet.mp. or Platelet Aggregation Inhibitors/ 4. clopidogrel.mp. or Clopidogrel/ 5. prasugrel.mp. or Prasugrel Hydrochloride/ 6. ticagrelor.mp. or Ticagrelor/ 7. 3 or 4 or 5 or 6 8. 1 and 2 and 7 9. limit 8 to medline records 10. limit 9 to embase records 11. 9 or 10 12. 12. 8 not 11 |
