# Supplementary material for: Short dual antiplatelet therapy duration after percutaneous coronary intervention in high bleeding risk patients: Systematic review and meta-analysis
Source: PLoS One. 2023 Sep 1;18(9):e0291061. doi: 10.1371/journal.pone.0291061 (PMC10473507; doi:10.1371/journal.pone.0291061)
Supplement: S1 Table — (DOCX) [file pone.0291061.s003.docx]

**S1 Table. Definition of high bleeding risk and proportion fitting each criterion within each trial**

|  | **MASTER DAPT^19^** | **TWILIGHT-HBR^23^** | **TICO-HBR^24^** | **STOPDAPT-2 Total Cohort-HBR^27^** | **STOPDAPT-2 -HBR^25,26^** |
| --- | --- | --- | --- | --- | --- |
| **HBR definition (% meeting criterion at baseline)** | ≥1 of the following criteria:   1. Age ≥75 years **(68.9%)**; 2. PRECISE-DAPT score ≥25 **(54.5%)**; 3. Clinical indication for treatment with oral anticoagulant (OAC) for at least 12 months **(36.9%)**; 4. Stroke at any time or transient ischemic attack in the previous 6 months **(9.6%)**; 5. Documented anemia, defined as repeated hemoglobin levels <11 g/dL or transfusion during the 4 weeks before inclusion **(11.4%)**; 6. Need for chronic treatment with steroids or NSAIDs **(8.8%)**; 7. Diagnosed malignancy (other than skin) considered at high bleeding risk including gastrointestinal, genitourethral/renal and pulmonary **(6.4%)**; 8. Recent (<12 months) nonaccess site bleeding episode(s) that required medical attention (i.e. actionable bleeding) **(4.3%)**; 9. Previous bleeding episode(s) that required hospitalization if the underlying cause had not been definitively treated **(4.1%)**; 10. Systemic conditions associated with an increased bleeding risk or any known coagulation disorder associated with increased bleeding risk **(1.8%)** | Modified ARC-HBR  **Major Criteria**   - Moderate/severe anemia **(24.2%)** - Severe/end-stage CKD **(6.9%)** - Thrombocytopenia **(5.1%)** - Liver disease **(2.2%)** - Prior major bleeding **(0.3%)**     **Minor Criteria**   - Moderate CKD **(55.4%)** - Age ≥75 years **(49.4%)** - Mild anemia **(43.3%)** - Long-term use of oral NSAIDs **(20.3%)** | ARC-HBR | PARIS bleeding score | Modified ARC-HBR  **Major criteria**   - Severe anemia **(25.0%)** - Severe CKD **(15.8%)** - Thrombocytopenia **(2.9%)** - Long-term anticoagulation **(1.2%)** - Liver cirrhosis **(1.0%)** - Previous hemorrhagic stroke **(0.8%)**   **Minor criteria**   - Age ≥75 years **(67.2%)** - Moderate CKD **(56.5%)** - Prior bleeding **(4.0%)** |
