# Supplementary material for: Short dual antiplatelet therapy duration after percutaneous coronary intervention in high bleeding risk patients: Systematic review and meta-analysis
Source: PLoS One. 2023 Sep 1;18(9):e0291061. doi: 10.1371/journal.pone.0291061 (PMC10473507; doi:10.1371/journal.pone.0291061)
Supplement: S1 Fig — (DOCX) [file pone.0291061.s004.docx]

**S1 Fig. Risk of bias assessment**


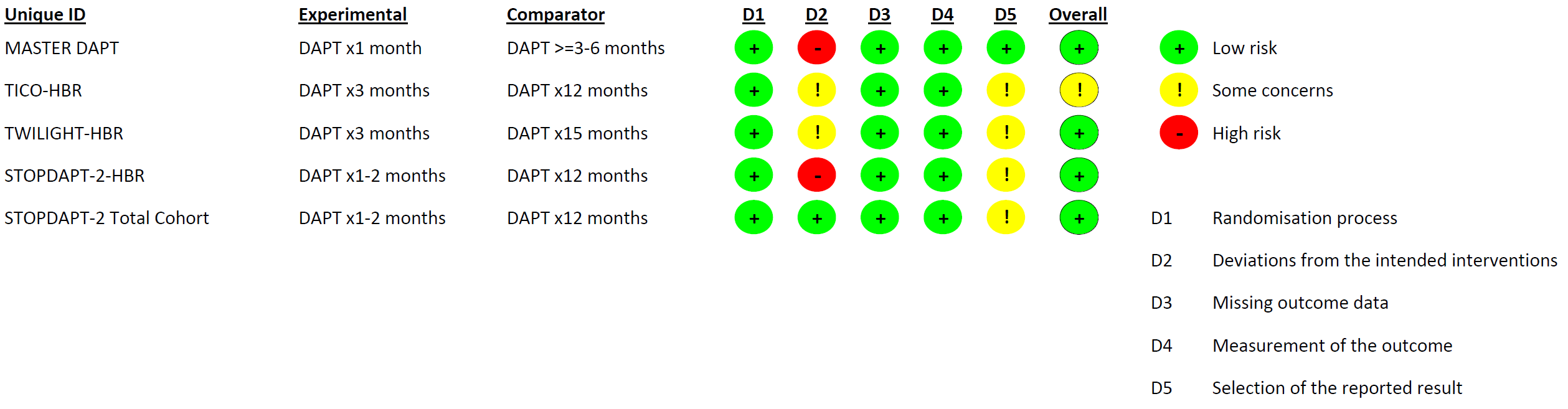


We rated the overall risk of bias for MASTER DAPT as low despite a rating of high risk of bias for domain 2. There were more deviations from study protocol in the intervention group (20.2% versus 9.4% non-adherence, Academic Research Consortium type 2/3), which could bias the results toward the null; however, the additional inverse probability-of-censoring weighting analysis suggested that any impact on outcomes would be minimal.

We rated the overall risk of bias for TICO-HBR to have some concerns. In the main TICO report, 12-14% did not adhere to assigned treatment (balanced between groups; domain 2). In addition, the HBR analysis was not pre-specified (domain 5).

We rated the overall risk of bias for TWILIGHT-HBR as low. There was some concern for risk of bias in domain 2 for MACE, as this outcome was only analyzed using the per-protocol population; however, a separate individual-patient-level meta-analysis including results from the overall TWILIGHT trial suggested that the intention-to-treat and per-protocol results for MACE were nearly identical. In addition, the HBR analysis was not pre-specified (domain 5).

We rated the overall risk of bias for STOPDAPT-2-HBR as low despite a rating of high risk of bias for domain 2. There were significant deviations from the study protocol in the intervention group; DAPT was stopped in only 10% during the first 30 days, but 95.2% during the first 60 days, while in the 12-month DAPT group, DAPT was maintained in 88.2% for 335 days. However, the result of this is that most participants in the intervention group received for DAPT for 2 instead of 1 months, which would still be considered as "short DAPT". In addition, the HBR analysis was not pre-specified (domain 5).

We rated the overall risk of bias for STOPDAPT-2 Total Cohort (which includes the aforementioned STOPDAPT-2 as well as STOPDAPT-2-ACS) as low. The HBR analysis was not pre-specified (domain 5), though this was not felt to impact the overall risk of bias.
