# Supplementary material for: Short dual antiplatelet therapy duration after percutaneous coronary intervention in high bleeding risk patients: Systematic review and meta-analysis
Source: PLoS One. 2023 Sep 1;18(9):e0291061. doi: 10.1371/journal.pone.0291061 (PMC10473507; doi:10.1371/journal.pone.0291061)

**S2 Figure. Subgroup based on acute coronary syndrome (ACS) versus non-ACS**

(A) MACE


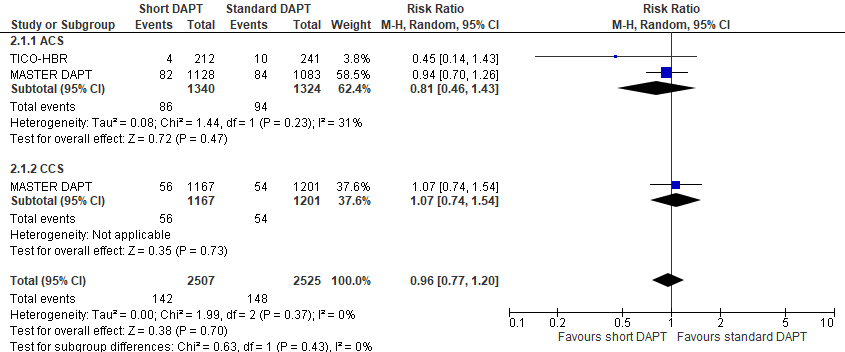


(B) All-cause death


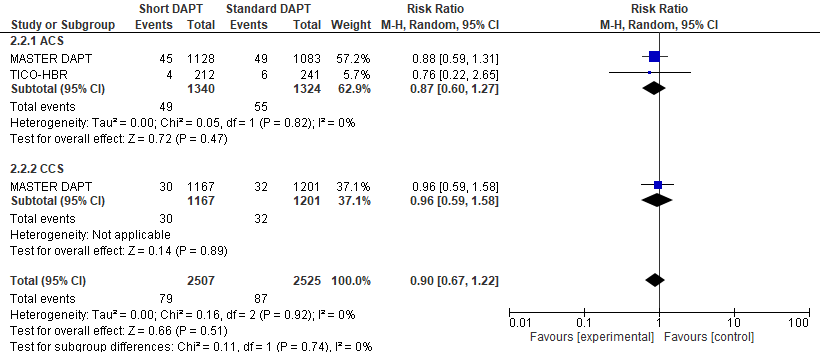


(C) Stent thrombosis


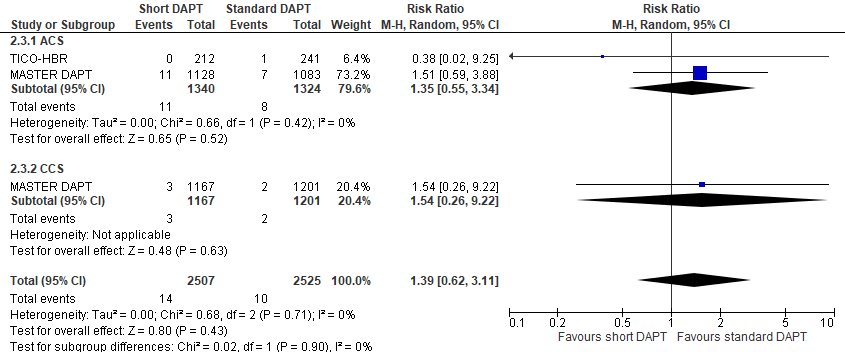


(D) Major bleed


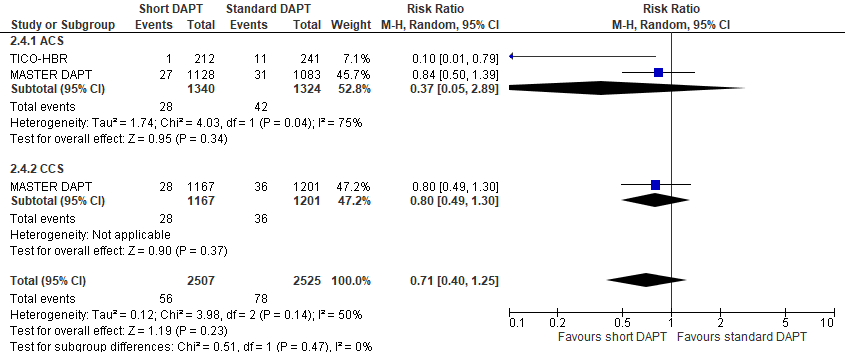


(E) Major or clinically-relevant non-major bleed


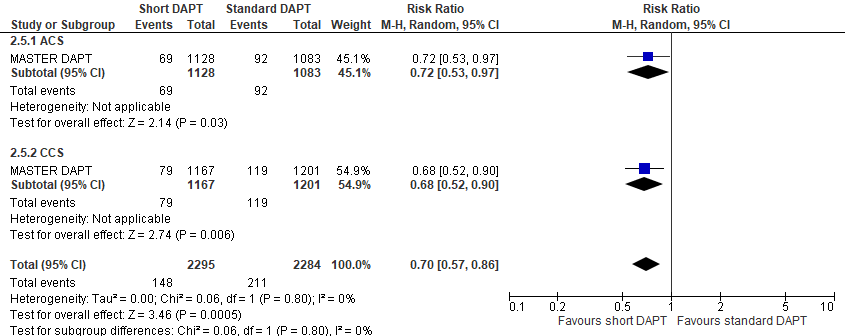

Supplement: S2 Fig — (DOCX) [file pone.0291061.s005.docx]
