# Supplementary material for: Short dual antiplatelet therapy duration after percutaneous coronary intervention in high bleeding risk patients: Systematic review and meta-analysis
Source: PLoS One. 2023 Sep 1;18(9):e0291061. doi: 10.1371/journal.pone.0291061 (PMC10473507; doi:10.1371/journal.pone.0291061)

**S4 Fig. Subgroup based on single antiplatelet therapy (SAPT) choice following dual antiplatelet therapy (DAPT) in the short DAPT arm**

(A) MACE


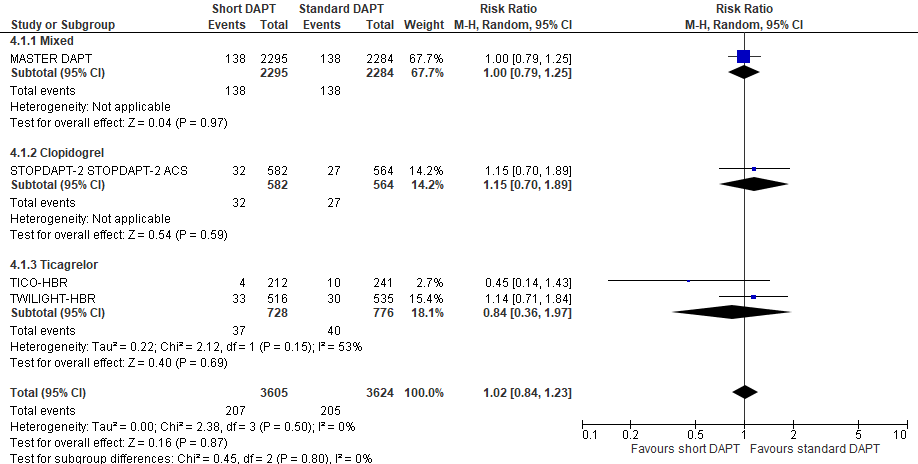


(B) All-cause death
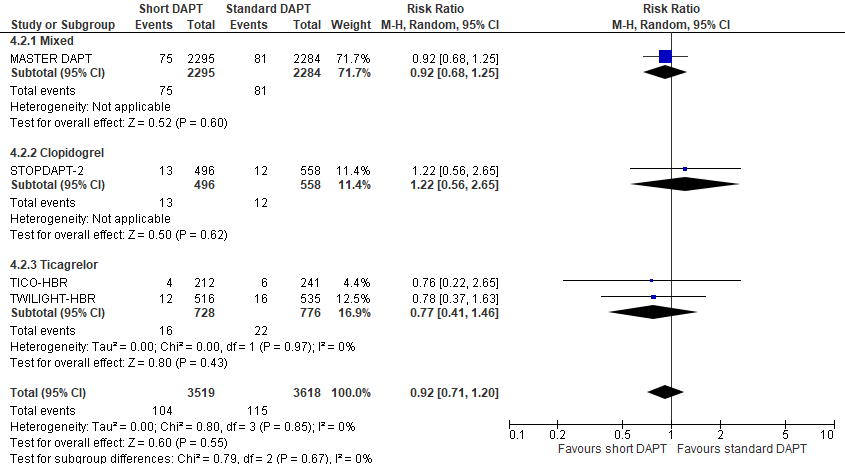


(C) Stent thrombosis
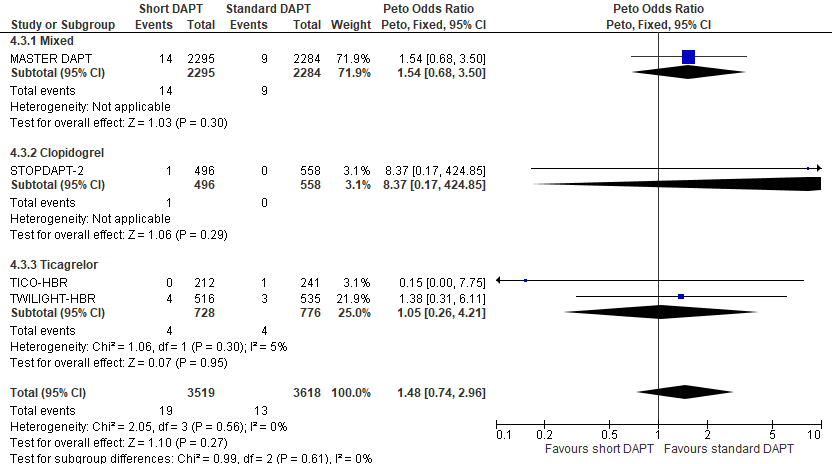


(D) Major bleed


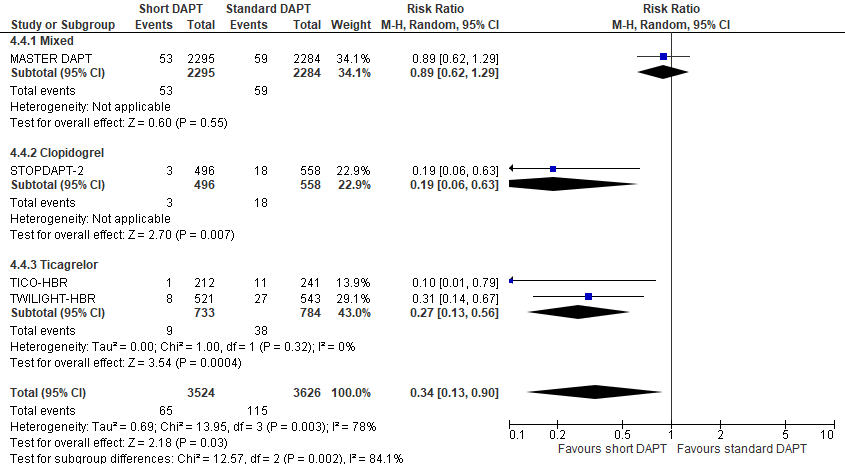


(E) Major or clinically-relevant non-major bleed


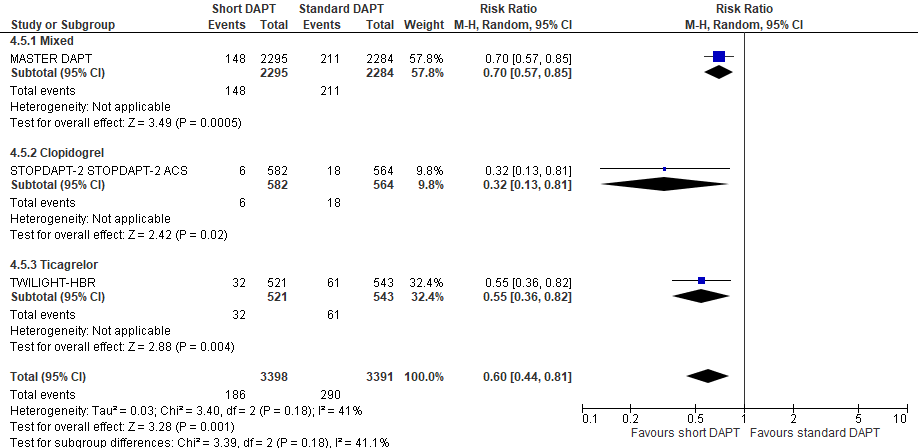

Supplement: S4 Fig — (DOCX) [file pone.0291061.s007.docx]
