# Supplementary material for: Short dual antiplatelet therapy duration after percutaneous coronary intervention in high bleeding risk patients: Systematic review and meta-analysis
Source: PLoS One. 2023 Sep 1;18(9):e0291061. doi: 10.1371/journal.pone.0291061 (PMC10473507; doi:10.1371/journal.pone.0291061)
Supplement: Attachment — Submitted filename: Response to Reviewers.docx [file pone.0291061.s008.docx]

**We thank the CJCO editors and reviewers for their thoughtful review. Below, we have itemized and addressed each comment and refer to the section within the manuscript that has been revised to address each comment.**

Reviewer #1:

**Item 1:** Figure S1 is not clear, there is some overlap between column 1 and 2.

**We thank the Reviewer for the opportunity to revise Supplemental Figure S1; we have removed the duplicate “study ID” column, as below:**

**Item 2:** Many studies on DAPT duration in HBR are non-randomized. I would understand their exclusion if you had included only RCT. However, this is not the case, as sub-analyses of previous RCT (possibly even non pre-specified) were included as well). How can a RCT have higher risk of bias in domain 2 than post-hoc analyses? Please consider reviewing.

**Thank you for the opportunity to clarify. We did only include RCTs, as observational studies are prone to bias, particularly selection bias for this clinical question. As the Reviewer notes, we included several sub-analyses of RCTs; however, these are different observational studies on this topic, as the included subgroup analyses still compared study interventions as randomized, and are therefore still classified as randomized comparisons.**

**For risk of bias assessment, we strictly adhered to the Cochrane Risk of Bias 2 guidance and signaling questions for all domains, which does not consider subgroup analysis as a source of significant source of bias (provided balanced baseline characteristics between groups within the subgroup). For MASTER DAPT, we rated domain 2 as high risk of bias due to imbalanced deviations from randomized treatment (20.2% versus 9.4% were non-adherent to their assigned treatment), which was also similarly an issue in STOPDAPT-2-HBR, but not the other included trials. However, it is important to note that we assigned an overall low risk of bias for the MASTER-DAPT trial despite this individual domain assessment.**

**To provide more clarity on why we assigned high risk of bias for domain 2 for the MASTER DAPT trial, we have revised the text within S1 Fig as follows:**

**“We rated the overall risk of bias for MASTER DAPT as low despite a rating of high risk of bias for domain 2. There were more deviations from study protocol in the intervention group (20.2% versus 9.4% non-adherence, Academic Research Consortium type 2/3), which could bias the results toward the null; however, the additional inverse probability-of-censoring weighting analysis suggested that any impact on outcomes would be minimal.”**

**Item 3:** You should provide definitions of bleeding and ischemic events. The fact that they are not consistent across studies needs to be taken into account.

**Table 1 summarize the outcomes included in each study’s major adverse cardiovascular events (MACE) composite outcome, as well as the standardized bleeding definition used. We also cite within the Methods sections the open-access article describing the ischemic/thrombotic event definitions (ref 16-17), as well as bleeding definitions (ref 18).**

**Item 4:** Publication bias should be assessed using funnel plots.

**Thank you for the opportunity to clarify this point. We planned to assess for publication bias by visual inspection of funnel plots for analyses with at least 10 trials because this test is considered to be of little value with fewer trials, per Cochrane methodology. Since all meta-analyses had fewer than 10 trials, we did not perform this analysis.**

**We have added the statement below to the revised manuscript:**

**Page 6, lines 102-104: “We planned to assess for publication bias by visual inspection of funnel plots for analyses with at least 10 trials because this test is considered to be of little value with fewer trials.[11]”**

**Item 5:** Why did you favored BARC bleeding definition over TIMI? Please comment and discuss.

***A priori*, we chose to prioritize the BARC criteria for bleeding events as this is a widely-used, universal and well-standardized categorization scheme. Additionally, major bleed by BARC criteria (3-5) is more inclusive and therefore sensitive than TIMI major bleeding, and better captures clinically-important major bleeding events missed by TIMI major bleed (e.g. bleed requiring transfusions or hospitalization but that have a hemoglobin drop <50 g/L).**

**We have revised the following section to address this comment:**

**Page 7, lines 111-112: “(BARC[18] classification [3 or 5 bleed] prioritized due to widespread use, and standardized, inclusive definitions”**

**Item 6:** I think a proper discussion on the literature is largely missing. There are several previous articles that should be cited and discussed in the paper. There is a wealth of papers on short DAPT, if one embark in another article in such topic, the discussion cannot ignore what has already been published, and most importantly should highlight why the work is different. Please refer to the following (which are a few but there are certainly more) and note that some of them are specific for patients at HBR:

1. doi: 10.1093/ehjcvp/pvaa127 - PMID: 33135064

2. doi: 10.1002/ccd.26110. Epub 2015 Aug 26 - PMID: 26309050

3. doi: 10.1093/ehjcvp/pvac065. Online ahead of print - PMID: 36427063

4. doi: 10.1093/ehjcvp/pvaa001 - PMID: 31942965

5. doi: 10.1093/eurheartj/ehac706 - PMID: 36477292

**We thank the Reviewer for sharing these references; we have incorporated references 1, 4, and 5 into our discussion and expanded the paragraph discussing our findings in context of previous evidence. We omitted reference 2 as it was an older [2016] review superseded by references 28 and 29 in our manuscript, and reference 3, which looked at a slightly different clinical question with different relevant trial eligibility than our review [triple antithrombotic therapy versus dual pathway therapy in patients with CAD and indication for an oral anticoagulant].**

**Page 14, lines 214-228: “The present study extends the results of prior systematic reviews that have focused primarily on patients without HBR undergoing PCI.[28–31] Consistent with our results, these previous systematic reviews found no further reduction in thrombotic events with standard or prolonged DAPT (≥12 months) compared with an abbreviated course followed by SAPT. Conversely, our study found a statistically significant and clinically meaningful reduction in major bleeding from reducing DAPT duration to 1-3 months in HBR patients, which has only inconsistently been observed in studies of non-HBR patients.[28–31] A recent meta-analysis of 9006 HBR patients – excluding patients with OAC – similarly found a reduction in bleeding events without compromising thrombotic outcomes.[32] Patients requiring an OAC have historically been excluded from prior DAPT studies, yet this is a key bleeding risk factor that impacts decisions around DAPT selection and duration.[13] The MASTER DAPT trial included in our review specifically included a subgroup of patients requiring OAC and stratified randomization according to indication for OAC.[22] In subgroup analyses within the present meta-analysis, bleeding reduction was greatest in patients without concomitant OAC; however, there was no increase in MACE with or without OAC indication.”**

**Item 7:** It would be really nice to summarize the main finding of the study in a central illustration. The paper lacks figures, which ensure clarity.

**We have added a central illustration figure as Figure 4, as below:**

Reviewer #2:

**Item 8:** The authors declared that the methodology outlined in the Cochrane Handbook for Systematic Reviews and Interventions, and reported following the 2020 PRISMA statement. However, the authors did not provide, among the others, the registration information for the review, nor report the reasons for the missing items in the limitation section.

**Thank you for the opportunity to clarify. We did not register our protocol (e.g. on PROSPERO or as a preprint). We have added a statement to this effect:**

**Page 5, lines 69-72: We performed a systematic review and meta-analysis according to the methodology outlined in the Cochrane Handbook for Systematic Reviews and Interventions using a prospectively-designed (but not registered) protocol, and reported following the 2020 PRISMA statement.[11] We wish to note here that prospective registration is not a PRISMA 2020 requirement, but reporting it is not registered is (which we now include).**

**We believe that we have now reported all PRISMA 2020 items, which is reflected in the attached PRISMA 2020 checklist (with details on where each item is reported).**

**Item 9:** Furthermore, the exclusion criteria (type of the analyses exclude as language, abstract presentation to congresses, and so on) were not detailed in the method section.

**We did not employ any exclusion criteria [i.e. all eligibility criteria were encompassed in our inclusion criteria: parallel randomized controlled trials (RCTs) that enrolled patients who underwent PCI for either ACS or non-ACS indications and had HBR, or reported on a subgroup of patients with HBR, and compared DAPT for 1-3 months followed by SAPT (“short DAPT”), to DAPT for 6-12 months (“standard DAPT”)].**

**Item 10:** Please report in the statistical section how the net clinical benefit (number of events for 1000 patients) has been assessed.

**We wish to clarify that we did not report net clinical benefit; the absolute differences per 1000 patients for each outcome reported in Table 2 were derived from the meta-analyses using GRADEPro. To clarify this, we have added the following:**

**Page 7, lines 118-119: “We further presented absolute differences using simple frequencies (per 1000 patients treated).”**

**Page 8, lines 124-125: ”** **We conducted all analyses using Review Manager version 5.4 (Cochrane, Copenhagen, Denmark) and GRADEPro.org.”**

**Item 11:** In order to look at the impact of each study on outcomes, the authors should perform an influence analysis at least for the primary outcome.

**We thank the Reviewer for this suggestion; however, we do not believe that influence analysis would add to the existing analyses, as there was no statistical heterogeneity for the outcomes of MACE, death and stent thrombosis, and existing analyses already identified MASTER DAPT as an outlier for the outcome of major bleeding (which subsequently could not be explained by our subgroup analyses).**

**For illustrative purposes, we provide the analyses for major bleeding with and without MASTER DAPT included in the pooled estimate below. In brief, the second figure provides a slightly more optimistic risk ratio point estimate, and narrower 95% confidence interval without the inconsistency from MASTER DAPT, but the results are overall consistent with the analysis that includes all studies.**

**Item 12:** It could also be interesting to perform a sensitivity analysis based on the type of platform used (resorbable versus non-resorbable polymer)

**As there was no heterogeneity for the outcomes of MACE, death, or stent thrombosis, this analysis does not demonstrate any differences between trials that used a biodegradable/resorbable stent (MASTER DAPT and TICO-HBR) compared with other stents (TWILIGHT-HBR and STOPDAPT-2 trials).**
