# Supplementary material for: Short dual antiplatelet therapy duration after percutaneous coronary intervention in high bleeding risk patients: Systematic review and meta-analysis
Source: PLoS One. 2023 Sep 1;18(9):e0291061. doi: 10.1371/journal.pone.0291061 (PMC10473507; doi:10.1371/journal.pone.0291061)
Supplement: Attachment — Submitted filename: Response to Reviewers2.docx [file pone.0291061.s009.docx]

**We thank the PLS ONE editors and reviewers for their thoughtful review. Below, we have itemized and addressed each comment and refer to the section within the manuscript that has been revised to address each comment.**

Reviewer #3: The authors performed a systemic review and meta-analysis of short DAPT vs. standard DAPT after PCI in patients with high bleeding risk. The following were my comments on this article:

1. The study population of these studies was not truly the entire group of patients with high bleeding risk after PCI. In TWILIGHT study, the eligible patients were required to be free from bleeding or ischemic events within the first 3 months after PCI. In TICO study, the key exclusion criteria included increased risk of bleeding due to prior traumatic brain injury or brain surgery within the past 6 months, internal bleeding within the past 6 weeks, and anemia. In STOPDAPT-2 study, the patients who experienced ischemic or bleeding complications during hospital stay post-PCI were excluded. In Master DAPT, the exclusion criteria included any active bleeding requiring medical attention (BARC > 2) within the first month after PCI. And the eligible patients were required to be free from adverse cardiovascular events during the first month after the index PCI. However, most bleeding complications after PCI occur within the first month of DAPT after PCI (doi:10.3390/jcm9061657). So these studies and the result of this meta-analysis could not apply to all patients with high-risk bleeding. On the contrary, they only represented some relatively “stable” high-risk patients after PCI. Patients with high bleeding risk could consider shortening the duration of DAPT and switching to P2Y12 inhibitor monotherapy if they do not have any ischemic or bleeding events within the first 1-3 months after PCI. The title and conclusion may mislead the readers.

**We thank the reviewer for this comment. We agree with them that this study does not apply to patients who have actively bled or experienced a subsequent ischemic event during the course of receiving DAPT; however, those patients represent 2 distinct patient groups/scenarios outside the scope of this study. We therefore respectfully disagree that the title or conclusion would mislead readers, particularly as we have used accepted definitions of high bleeding risk (HBR), the primary patient population of this trial, for eligibility into this study (Academic Research Consortium HBR definition and analogous/modified versions of this definition).**

2. The subgroup analysis for ACS vs CCS in patients with HRB had many questionable issues. First, the compare group in Master DAPT might receive suboptimal DAPT for ACS patients after PCI, because all ACS patients in compare group received only 6-months DAPT. SMART-DATE trial (https://doi.org/10.1016/S0140-6736(18)30493-8) had proved that shortening the duration of standard 12-month DAPT to 6-month DAPT in ACS patients would significantly increase the risk of recurrent ischemic events. Second, they were only two trials in this subgroup analysis. Third, STOPDAPT-2 ACS study had failed to attest noninferiority to standard 12-months of DAPT for the net clinical benefit with a numerical increase in cardiovascular events. Fourth, there was a similar observation in TWILIGHT-HBR subgroup analysis. The rate of all-cause death, MI, or stroke was increased in ticagrelor monotherapy (6.5% vs. 5.6%; HR 1.16). When the patient number becomes larger, the number would be statistically significant.

**We agree with the reviewer that the ACS vs non-ACS subgroup analysis is limited by being reported for only a subset of the studies; however, the main finding remains that there is no heterogeneity of treatment effect explained by this subgroup. Addressing the specific points raised by the reviewer:**

- **In ACS patients, MASTER DAPT required a minimum of 6 months of DAPT in the control arm; however, most patients received a duration closer to 12 months if they were not receiving an oral anticoagulant at baseline (in which case they would have received dual-pathway therapy). The following figure is from the MASTER DAPT complex PCI subgroup (in which ACS was categorized). Dark blue indicates patients receiving DAPT. We further note that both current American and European guidelines advocate for a reduced DAPT duration of 6 months in ACS patients treated with PCI in HBR patients (prior to the emergence of this evidence). Therefore, we disagree that the comparator arm of MASTER DAPT was suboptimal.**
  -

- **We acknowledge the results of SMART-DATE; however, we note that other studies comparing 6 to 12 months of DAPT after PCI in non-HBR patients did not find this difference in ischemic events (ref 28 from our manuscript). In either case, these results are not directly applicable to the HBR patients who are the target population of our study.**
- **The authors note that non-significant differences within individual studies (“trending” toward harm with shorter DAPT) would be statistically significant with a larger sample size, though this did not emerge in our meta-analyses and unlikely based on theoretical and simulation grounds (**[**https://www.bmj.com/content/bmj/348/bmj.g2215.full.pdf**](https://www.bmj.com/content/bmj/348/bmj.g2215.full.pdf)**).**

3. The duration of DAPT and the choice of subsequent SAPT(aspirin or specific P2Y12 inhibitors) need to take into discussion together. Since most successful clinical trials were applying P2Y12 inhibitor monotherapy after shortened DAPT, it is not appropriate to mix the results of aspirin monotherapy with P2Y12 inhibitor monotherapy. (Most patients in Master DAPT trial were also receiving P2Y12 inhibitor monotherapy after 1-month DAPT)

**We agree that discussion of which antiplatelet should be retained following DAPT is important, and addressed this in sensitivity analyses (S4 Fig). However, MASTER DAPT was the only trial that allowed ASA monotherapy, and available manuscripts did not disaggregate ASA from P2Y12i use, so this trial was classified as “mixed”.**

**We discuss this in paragraph 3 of the discussion: “It remains unclear which antiplatelet agent would be best for SAPT following short DAPT. Most patients in the present study received a P2Y12 inhibitor (clopidogrel or ticagrelor) following short DAPT. Additional studies suggest that a P2Y12 inhibitor may be superior to acetylsalicylic acid monotherapy following DAPT. The HOST-EXAM trial conducted in South Korea found that clopidogrel 75 mg daily reduced the risk of MACE and major bleeding compared with ASA 100 mg daily after 6-18 months of DAPT in DES PCI.[35] Additionally, the CAPRIE trial had previously demonstrated lower risk of MI and bleeding with clopidogrel 75 mg daily compared with ASA 325 mg daily.[36]”**

**In addition to these existing elements, we have added the following to the first (summary) paragraph of the discussion, lines 213-214: “Finally, these results were consistent regardless of clinical presentation, concomitant indication for OAC, or choice of SAPT following DAPT, though most patients received P2Y12 inhibitor monotherapy.”**

4. Since this article is a “systemic review and meta-analysis”, I expect the paragraph of “discussion” and “limitation” should be more words and discussion. The gap between current evidence from previous studies and clinical practice could be considered to add into your discussion.

**We thank the reviewer for this comment; however, we note that the discussion addresses differences from existing evidence (both in the overall population of patients requiring DAPT, as well as the subgroup of HBR patients), and contrasts to current practice in paragraphs 2 and 3, respectively, as well as addresses other practical elements (which antiplatelet to use as stepdown after DAPT). We have therefore elected to retain the currently succinct discussion.**

5. In Table 1. “STOPDAPT-2 Total cohort-HBR” and “STOPDAPT-2 HBR” were presented as separate trials. Does ”STOPDAPT-2 total cohort (STOPDAPT-2 + STOPDAPT-2 ACS)-HBR” include the patients enrolled in “STOPDAPT-2 HBR”? In my understanding, “STOPDAPT-2 Total cohort-HBR” and “STOPDAPT-2 HBR” should be viewed as ONE trial.

**We thank the reviewer for the opportunity to clarify. STOPDAPT-2 and STOPDAPT-2 ACS were two distinct trials, with HBR data reported in two separate publications: The STOPDAPT-2 Total cohort-HBR manuscript (both trials) and the STOPDAPT-2 HBR manuscript (only STOPDAPT-2). Although there is overlap in the patient populations between these two manuscripts, we included both within our study (and reported the two substudies separately in Table 1), as they present distinct results, including reporting on different subsets of our outcomes of interest. We indicate in the text and forest plots for each outcome whether the outcomes were reported in both STOPDAPT studies (total cohort-HBR) or the STOPDAPT-2-HBR manuscript (e.g. “All trials except STOPDAPT-2 ACS reported on all-cause death and stent thrombosis”).**

**To further clarify this point,**

- **We have added the following footnote to Table 1: “*STOPDAPT-2 Total Cohort-HBR included pooled HBR subgroup data from two distinct trials: STOPDAPT-2 and STOPDAPT-2 ACS, whereas STOPDAPT-2-HBR included HBR subgroup data for STOPDAPT-2 only (i.e. not STOPDAPT-2 ACS).”**
- **We have revised the following sentence in the Results, lines 136-139: “The included studies (and subgroup analyses) were: MASTER DAPT (the only RCT exclusively enrolling patients with HBR), [19–22] and the HBR subgroup of TWILIGHT[23], TICO[24], and STOPDAPT-2 Total Cohort (which consisted of two trials: STOPDAPT-2 and STOPDAPT-2 ACS trials)[25–27]”**

6. In Figure 2C, It would be better if the order of each included study could be the same as the order used in most of your analyses. So the “TICO-HBR” should be the first one, and “STOPDAPT-2” be the second one.

**Please note that for all forest plots in the present manuscript, including Figure 2c, trials are deliberately ordered by statistical weight (ascending) to illustrate the relationship between the pooled effect size and the largest trial. We have retained this in the present revision.**
